# Supplementary material for: A Q fever outbreak associated to courier transport of pets
Source: PLoS One. 2019 Nov 25;14(11):e0225605. doi: 10.1371/journal.pone.0225605 (PMC6876792; doi:10.1371/journal.pone.0225605)
Supplement: S1 Appendix — (DOCX) [file pone.0225605.s001.docx]

| **PATIENT DATA** |
| --- |

Name and surname:

Date of birth: __/__/___ Age (years): ____

Gender: Male  Female  Nationality: _________________

Residency address:

Country: _______________ Region: ___________________

Province: ___________ City/Village: Postal code:

Address: _________________________________________________ Phone: _______________

Is this a rural area? _______________________

Do you have any animals at home? (if yes, describe) _____________________

Local Health Service Centre: ______________________________

Name of GP: ___________________________

| **DISEASE DATA** |
| --- |

Date of first symptoms: ___ /___ /_____

Description of symptoms (mark YES, if you suffered any of the following symptoms or NO if you did not):

Fever Yes  No

Headache Yes  No

Shivering Yes  No

Malaise Yes  No

Myalgia Yes  No

Pneumonia Yes  No

Expectoration Yes  No

Vomiting Yes  No

Diarrhoea Yes  No

Hepatitis Yes  No

Other _____________

Indicate if you received medical care of any of the following types:

Hospitalization: No  Yes

If your answer is yes, indicate hospital name and dates _____________

Emergency services: No  Yes

If your answer is yes, indicate Centre name and dates __________________________

Primary health care services: No  Yes

If your answer is yes, indicate Centre name and dates __________________________

Was any Q fever specific test performed when you received medical attention as stated above?

No  Yes  If your answer is yes, indicate Centre name __________________________

Was a diagnostic X ray performed when received medical attention?

No  Yes  If your answer is yes, indicate Centre name __________________________

| **OCCUPATIONAL DATA** |
| --- |

Name and address of the employer company:

Working area:

How do you get to work? ___________________________________________________________

Do you go out for lunch during the working day? __________ If yes, how do you get to the restaurant/eating place?

Do you go out to smoke during the working day?

Within the 30 days before getting infected, did you carry out any tasks at work that involved contact with pets or other animals? No  Yes  If you answered yes, please, indicate the type of animals and dates:

Cats  Dates ______________

Dogs  Dates ______________

Miniature goats  Dates ______________

Other  Specify ________________ Dates ________________

Describe type of contact with the animals:

Direct contact:

Handling, exploration or cleaning the animals

Handling of animal feces or other animal discharges

Indirect contact:

Receipt, transport, deposit or delivery of the container while the animal was inside

Cleaning of the containers

Getting close to the transport containers or the pet holding site

Other (specify) _____­­­­­­­­­­­­­­­­­­­­_____

Specifically, did you get in contact with animals at work in the period between June 1 and August 8? No  Yes  If the answer is yes, specify___________________________

| **EXPOSURE** |
| --- |

Within the 30 days before getting infected, did you live in a farm or visited a farm or any other premises with animals? No  Yes  If your answer is yes, please, specify:

Location and dates ________________________________________________________________

Type of animals __________________________________________________________________

Type of contact ___________________________________________________________________

Any recent parturitions in that farm/animal premises: Yes  No  Unknown , if your answer is yes, please, explain ___________________________

Are there any animals/farms in the surroundings of your home or working place? No  Yes  If your answer is yes, please, specify:

Type of animals __________________________________________________________________

Did you get in direct contact with those animals? No  Yes  If your answer is yes, please, specify type of contact _______________________________________________

Any recent parturitions among those animals? No  Yes  Unknown , if your answer is yes, please, explain ___________________________

Within the 30 days before getting infected, did you walk or ride a bicycle around areas with animals? No  Yes  If your answer is yes, what type of animals? _________________________________

Did you get in direct contact with those animals? No  Yes

Within the 30 days before getting infected, did you drink milk or ate unpasteurized dairy products? No  Yes  If your answer is yes, please, specify:

Type of product __________________________

Date of consumption ______________________

Did you travel during the last 30 days?

No  Yes , if your answer is yes, specify the dates________

| **RISK ASSOCIATED DATA** |
| --- |

Do you know anyone near you who has recently suffered from pneumonia, fever and/or hepatitis?

No  Yes , if your answer is yes, specify:

Pathology____________________________________

Name and surname________________________________________________

Telephone _________

Relationship____________________

Do you suffer from any disease or are you taking an immunosuppressing treatment?

No  Yes , if your answer is yes, specify ______________________________________________

Do you have a valvula phrosthesis or do you suffer a heart valves disease or any other vascular disease? No  Yes , if your answer is yes, specify ______________________________

Are you pregnant? No  Yes , specify weeks of pregnancy___________

**Date when the survey was filled in** --/---/-- **Name of the worker**______________

**COMMENTS**^^[[1]](#footnote-1)^^

1. Include any relevant information not mentioned in the questionnaire [↑](#footnote-ref-1)
